# Supplementary material for: Comparative genomics provides new insights into the diversity, physiology, and sexuality of the only industrially exploited tremellomycete: Phaffia rhodozyma
Source: BMC Genomics. 2016 Nov 9;17:901. doi: 10.1186/s12864-016-3244-7 (PMC5103461; doi:10.1186/s12864-016-3244-7)
Supplement: Additional file 6: — List of orphan genes with links to PFAM (related to Additional file 1: Table S1). (ZIP 1428 kb) [file 12864_2016_3244_MOESM6_ESM.zip › PFAM_UNIQUE/G02098_P.html]

#                                                                            --- full sequence --- -------------- this domain -------------   hmm coord   ali coord   env coord
  
# target name        accession   tlen query name           accession   qlen   E-value  score  bias   #  of  c-Evalue  i-Evalue  score  bias  from    to  from    to  from    to  acc description of target
  
#------------------- ---------- ----- -------------------- ---------- ----- --------- ------ ----- --- --- --------- --------- ------ ----- ----- ----- ----- ----- ----- ----- ---- ---------------------
  
G02098\_P             -            728 BicD                 PF09730.4    717       7.3    3.4  13.4   1   1     0.055        13    2.7  13.4   299   419    48   166    31   209 0.63 - Microtubule-associated protein Bicaudal-D  
G02098\_P             -            728 Cytochrom\_C552       PF02335.10   435       4.5    4.5   6.6   1   1    0.0037       7.4    3.8   6.6   261   330    97   169    85   197 0.82 - Cytochrome c552  
G02098\_P             -            728 DMAP1                PF05499.7    176       0.7    8.1  10.9   1   2   7.2e-05      0.43    8.8   2.4     6    90   105   195   102   202 0.65 - DNA methyltransferase 1-associated protein 1 (DMAP1)  
G02098\_P             -            728 DMAP1                PF05499.7    176       0.7    8.1  10.9   2   2    0.0025        15    3.8   1.5    13    47   273   308   262   318 0.65 - DNA methyltransferase 1-associated protein 1 (DMAP1)  
G02098\_P             -            728 DUF3702              PF12486.3    148       5.3    5.4   7.8   1   1    0.0052        16    3.9   7.8    97   134   107   145   106   150 0.85 - ImpA domain protein  
G02098\_P             -            728 Fib\_alpha            PF08702.5    146       3.9    6.2   6.8   1   1     0.038        13    4.5   6.8    79   122    97   139    44   176 0.63 - Fibrinogen alpha/beta chain family  
G02098\_P             -            728 FTA4                 PF13093.1    213       3.2    5.8   8.4   1   1    0.0046       6.9    4.7   8.4   109   166    48   141    32   169 0.76 - Kinetochore complex Fta4 of Sim4 subunit, or CENP-50  
G02098\_P             -            728 RNA\_pol\_Rpc82        PF05645.8    258       6.8    4.8   7.0   1   2   0.00088       1.8    6.7   3.1   155   222    86   168    59   193 0.67 - RNA polymerase III subunit RPC82  
G02098\_P             -            728 RNA\_pol\_Rpc82        PF05645.8    258       6.8    4.8   7.0   2   2      0.51     1e+03   -2.3   0.1   201   201   358   358   311   428 0.49 - RNA polymerase III subunit RPC82  
G02098\_P             -            728 Spc7                 PF08317.6    326       6.2    4.0   7.0   1   2      0.72   1.3e+02   -0.3   6.0   209   271   103   165    33   190 0.69 - Spc7 kinetochore protein  
G02098\_P             -            728 Spc7                 PF08317.6    326       6.2    4.0   7.0   2   2     0.083        15    2.8   0.0   145   172   458   485   457   504 0.84 - Spc7 kinetochore protein  
G02098\_P             -            728 T2SJ                 PF11612.3    169       3.2    6.1   8.7   1   2    0.0044        27    3.1   6.7     9    89    96   175    90   196 0.73 - Type II secretion system (T2SS), protein J  
G02098\_P             -            728 T2SJ                 PF11612.3    169       3.2    6.1   8.7   2   2     0.011        64    1.9   0.0    13    40   466   495   464   529 0.75 - Type II secretion system (T2SS), protein J
